# Serum Matrix Metalloproteinase-7 is an independent prognostic biomarker in advanced bladder cancer

**DOI:** 10.1186/s40169-014-0031-4

**Published:** 2014-10-28

**Authors:** Mounira El Demery, Gaiané Demirdjian-Sarkissian, Simon Thezenas, William Jacot, Yassine Laghzali, Bruno Darbouret, Stéphane Culine, Xavier Rebillard, Pierre-Jean Lamy

**Affiliations:** 1 Department of Medical Oncology, Cap d¿Or Clinic, La Seyne sur Mer, France; 2 Clinical Diagnostic Division, Thermo Fisher Scientific, Nimes, France; 3 Department of Biostatistics, Institut Régional du Cancer de Montpellier (ICM), Val d¿Aurelle, Montpellier, France; 4 Department of Medical Oncology, Institut Régional du Cancer de Montpellier (ICM), Val d¿Aurelle, Montpellier, France; 5 Hôpital Saint-Louis, APHP, Saint-Louis, France; 6 Clinique Beausoleil, Montpellier, France; 7 Department of Biology and Oncogenetics, Institut Régional du Cancer de Montpellier (ICM), Val d¿Aurelle, Montpellier, 34298, France

**Keywords:** MMP-7, Bladder cancer, Prognosis, Urothelial tumors, Serum, Biomarker, Tumor markers, Overall survival

## Abstract

**Background:**

Urine markers have been studied extensively but there is a lack of blood prognostic markers in bladder cancer. MMP-7 is produced by stromal cells and by tumor cells and is overexpressed in a variety of epithelial and mesenchymal tumors. In this study, we assessed with an immunoassay we developed, the prognostic value of serum MMP-7 in a series of patients with advanced bladder cancer.

**Methods:**

Serum samples were collected from 56 patients with advanced bladder cancer who were treated at the Montpellier Cancer Institute between March 2003 and December 2004. MMP-7 was quantified in serum samples by using a homogeneous sandwich fluoroimmunoassay we developed based on the time resolved amplified cryptate emission (TRACE) technology.

**Results:**

The median overall survival of the study population was 2.2 years (95% CI, 1.4 to 3.0) with 1- and 5-year survival rates of 73% (95% CI, 59% to 82%) and 25% (95% CI, 14% to 37%), respectively. High MMP-7 serum levels were associated with poor survival. Using a cut-off value of 11.5 ng/mL, the median overall survival was 3.0 years (95% CI, 1.5 to 5.1) for patients with MMP-7 serum level <11.5 ng/mL and 1.3 years (95% CI, 0.8 to 2.5) for patients with serum level ?11.5 ng/mL. Multivariate analysis identified high MMP-7 serum concentration as an independent prognostic factor for survival in patients with advanced bladder cancer (R?=?2.1, 95% CI, 1.1 to 4.4).

**Conclusions:**

Our results show that the MMP-7 serum concentration is an independent prognostic factor in patients with locally advanced and or metastatic bladder cancer.

## Background

Bladder urothelial cell carcinoma (BUCC) is the fourth most common cancer in men and overall the fifth most common cancer with 11965 new cases and 4772 BUCC-related deaths in France in 2012 [1]. At diagnosis, approximately 75% of bladder cancers are non-muscle invasive [pTa, pT1, carcinoma in situ (CIS)], 25% are muscle-invasive (pT2-pT4) and 5% are metastatic (N1-3 or M1) [2]. The standard treatment includes transurethral resection of the bladder tumor (TURBT) and intravesical chemotherapy or immunotherapy for superficial tumors and radical cystectomy for non muscle invasive tumors. However, 50-70% of patients will experience tumor recurrence within 5 years. The 5-year survival rate of patients with non-muscle invasive disease is between 95% (Ta tumors) and 75% (T1 tumors), and almost 25% of patients with a Ta-T1 tumor will eventually develop invasive disease [3]. The 5-year survival rate further decreases in patients with muscle invasive or metastatic disease (60% and 35% for T2 and T3 tumors, respectively, and only 10% for T4 tumors) [4]. The bladder cancer heterogeneity in terms of clinical behavior means that prognostic tools based on clinical and pathological variables are not accurate enough to reliably predict the tumor biological behavior or to guide treatment choices. Urine markers have been studied extensively but cannot be easily standardized due to diuresis variations, and blood prognostic markers for bladder cancer are lacking. New markers to aid in the diagnosis and prognosis as well as to identify the optimal treatment and monitor tumor progression are therefore needed [5].

Matrix Metalloproteinases (MMPs) are a family of zinc-dependent proteolytic enzymes that cleave extracellular matrix proteins (ECMs). ECM degradation is an important step in both physiological (embryonic development, reproduction, wound healing) and pathological processes (arthritis, tumor progression and metastasis formation) [6]. Furthermore, MMPs can influence several molecular processes involved in tumor progression through their ability to cleave pro-apoptotic factors, cell surface molecules, cell adhesion molecules and growth factors [7],[8]. MMPs can mobilize pro-angiogenic inhibitors, such as endostatin and angiostatin [9],[10]. MMP-7, also known as matrilysin, is the smallest MMPs; its molecular weight as a proenzyme is 28 KDa and it decreases to 19 KDa after activation induced by plasmin and trypsin [11]. MMP-7 is expressed in the ductal and glandular epithelia of normal mammary and parotid glands, liver, pancreas and prostate. In human tumors, MMP-7 is produced by stromal cells (macrophages, fibroblasts and endothelial cells) and also by tumor cells [6]. MMP-7 is overexpressed in a variety of epithelial and mesenchymal tumors, such as esophageal, colon, liver, renal and pancreatic cancer [12]-[14]. Furthermore, increased circulating levels of MMP-7 have been correlated with metastatic disease and poor patients¿ survival in colorectal, ovarian and renal cancer [15]-[18]. Elevated MMP-7 concentration was detected in urine samples from patients with regional or distant metastasis in comparison to patients with localized disease and controls, suggesting that MMP-7 could be a putative biomarker for the diagnosis and monitoring of bladder cancer [19]. Moreover, MMP-7 plasma levels are significantly higher in patients with bladder cancer at high risk of diseases progression [20]. Similarly, analysis of MMP-7 plasma level in 135 patients with localized bladder cancer (?T1) confirmed the significant association of MMP-7 level with cancer-related death [21].

In the present study, we measured MMP-7 serum levels particularly in patients with advanced bladder cancer and analyzed their correlation with clinical parameters to determine whether serum MMP-7 could also be used as a prognostic marker.

## Methods

### Clinical samples

Serum samples from 56 patients with bladder cancer were collected in the Department of Medical Oncology at the Institut du Cancer de Montpellier (ICM) between March 2003 and December 2004 and stored in a certified AFNOR 96900 biobank (ICM biobank number: **BB-0033-00059)**. The patients¿ medical records were used to extract information on the clinico-pathological features, last follow-up and cause of death. This study was performed in compliance with the relevant French ethical standards and was validated by our Institutional Research Ethics Board (CORT). According to our biobank policy all patients gave their general consent for the use of their biological samples in research.

### Measurement of MMP-7 serum level

We previously developed and validated a homogeneous sandwich fluoroimmunoassay [18] based on the TRACE technology. The specific MMP-7 immunoassay was set up using two monoclonal anti-human MMP-7 antibodies (29S and 01S) generated by immunization of female BALB/c mice with human MMP-7 recombinant protein (R&D Systems Europe Ltd., Abingdon, UK). The immunoassay was performed by incubating 50 ?L of each patient¿s serum sample or calibrator with 50 ?L of AF647-conjugated 29S-antibody solution) and 50 ?L of) cryptate-conjugated 01S-antibody solution at 37°C in a BRAHMS KRYPTOR automate (Thermo Fisher Scientific, Hennigsdorf, Germany), according to the manufacturer¿s instructions and as previously described [18]. Results were given in ?g/L. Standards ranged from 0.5 to 40 ?g/L.

### Statistical analysis

Categorical variables were reported using contingency tables. For continuous variables, median and range were computed. To investigate their associations with the clinical, pathological and biological parameters, univariate statistical analyses were performed using the Pearson¿s chi-square test or the Fisher¿s exact test, when applicable, for categorical variables, and using the Kruskal-Wallis test or the Student¿s t test for continuous variables. Survival times were measured from the date of the diagnosis of bladder cancer to the event date (or to the last available follow-up, if not applicable). Overall survival rates (with event defined as death from any cause) were estimated according to the Kaplan-Meier method and presented with 95% confidence intervals (CIs). Patients alive were censored at the date of the last follow-up. The median follow-up was estimated according to the « reverse Kaplan-Meier method » and presented with 95% CIs. Survival curves were drawn and the log-rank test was performed to assess differences between groups. Multivariate analyses were carried out using Cox proportional hazards regressions with a stepwise selection procedure to investigate known prognostic factors. Hazards Ratios (HR) with 95% CIs show the risk of experiencing the event (death from any cause) during the follow-up period. All reported P values are two-sided and the significance level was set at 5% (p?<?0.05). Statistical analyses were performed using the STATA 11 software *(Stata Corporation, College Station, TX).*

## Results

### Clinical background

Patient¿s characteristics at time of diagnosis are shown in Table 1. A total of 56 patients who received a diagnosis of urothelial tumor between January 1995 and November 2004 were enrolled in this study. At the time of the analysis, 35/56 patients (62.5%) had metastatic disease and 20 patients (35.7%) had localized disease. Seven patients (12.5%) had a Performance Status (PS) of 2/3. Forty-six (82.1%;) had grade 3 tumor at diagnosis. Surgery was performed for 18 (32.1%) patients; radical cystectomy for 13 (23.2%) patients and nephrectomy for 5 (8.9%) patients. Seventeen patients (30.4%) received post-operative chemotherapy and 12 (21.4%) concomitant radio-chemotherapy. The median follow-up was 8.1 years (range: 0.2- 16.2 years).

**Table 1:** Patients¿ characteristics

	**N°**	**%**
**Sex**		
????????M	47	83.9
????????F	9	16.1
**AGE** ???????Median (Range)	69 (49 - 91)	
**Histology**		
????????Urothelial	47	83.9
????????Other	3	5.4
????????Unknown	6	10.7
**TNM at diagnosis**		
**T**		
????????Tx	4	7.1
????????T2	2	3.6
????????T3	16	28.6
????????T4	34	60.7
**N**		
????????Nx	31	55.4
????????N0	12	21.4
????????N1	10	17.9
????????N2	3	5.4
**M**		
????????M0/Mx	46	82.1
????????M1	10	17.9
**Grade**		
????????Gr2	1	1.8
????????Gr3	46	82.1
????????Unknown	9	16.1
**PS**		
????????0	31	55.4
????????1	17	30.4
????????2	7	12.5
????????3	1	1.8
**Treatment**		
????????Surgery	18	32.1
????????Radio-chemotherapy	12	21.4
????????Chemotherapy	39	69.6
????????Radiotherapy	1	1.8
????????Palliative care	3	5.4
**MMP7 (ng/mL)**		
????????Mean (s.d.)	10.6 (8.8)	
????????Median (range)	8.5 (2.8-44.2)	

### MMP-7 serum concentration and survival:

**Figure 1:**
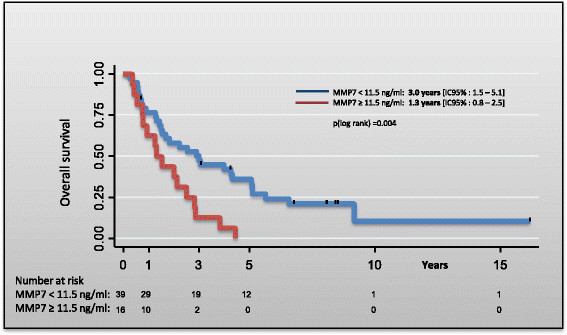
Overall survival probability function according to serum MMP-7 level in bladder cancer.

The median overall survival of the study population was 2.2 years (95% CI 1.4- 3.0) with 1- and 5-year survival rates of 73% (95% CI 59-82%) and 25% (95% CI 14-37%), respectively. Among the 56 patients, 47 died (38 patients: cancer-related death; 9 patients: non-cancer related death). MMP-7 serum levels were categorized in deciles and then the effect of MMP-7 serum level on the survival time was assessed (univariate Cox regression analysis). MMP-7 serum levels higher than 11.5 ng/mL were significantly associated with poor prognosis (Table 2). Therefore, 11.5 ng/mL was used as cut-off to classify patients as low (<11.5 ng/mL serum MMP-7) and high risk (?11.5 ng/mL serum MMP-7) for the univariate (Table 3) and multivariate analysis (Table 4). The median overall survival of patients with MMP-7 serum level <11.5 ng/mL and those with serum level ?11.5 ng/mL was 3.0 years (95% CI 1.5-5.1) and 1.3 years (95% CI 0.8-2.5), respectively (Figure 1); p(log rank)?=?0.004 (Table 1). Multivariate analysis indicated that MMP-7 serum level ?11.5 ng/mL was an independent predictor of poor survival (Table 4).

**Table 2:** Univariate cox regression analysis using MMP-7 categories (deciles)

**MMP7 category(ng/mL)**	**HR (95% CI)**	**P**
**<3.6**	**1.0**	**-**
[3.6 ¿ 4.2]	0.4 (0.1-0.2)	0.296
[4.2 ¿ 5.2]	2.9 (0.7-12.1)	0.126
[5.2 ¿ 7.2]	1.5 (0.4-5.8)	0.493
[7.2 ¿ 8.4]	4.8 (1.2-19.5)	0.026
[8.4 ¿ 9.6]	1.2 (0.3-5.7)	0.789
[9.6 ¿ 11.5]	0.7 (0.2-3.0)	0.682
[11.5 ¿ 13.4]	5.4 (1.3-23.6)	0.022
[13.4 ¿ 17.5]	3.4 (0.9-13.1)	0.072
? 17.5	3.7 (0.9-15.5)	0.067

The MMP7?11.5 ng/mL categories showed consecutive P<0.1 values.

**Table 3:** Univariate survival analyses

**Variable**	**HR [95%CI] **	**P**
Gender		
??Male	1.0	
??Female	0.75 [0.34 ; 0.63]	0.472
pN		
??pN0	1.0	
??pN+	2.33 [0.87 ; 6.24]	0.090
pM	pM	
??M0	1.0	
??M1	1.64 [0.74 ; 3.62]	0.225
Grade		
??2	1.0	
??3	0.95 [0.13 ; 7.00]	0.96
Hemoglobin		
??Normal	1.0	
??Abnormal	1.39 [0.76 ; 2.56]	0.29
PS		
??0-1	1.0	
??2-4	1.31 [0.58 ; 2.98]	0.51
MMP-7		
??<11.5 ng/mL	1.0	
??>=11.5 ng/mL	2.55 [1.33 ; 4.90]	0.005

**Table 4:** Multivariate survival analysis

		**HR (CI 95%)**	**P**
**Gender**	Male	1.0	
	Female	0.7 (0.3-1.6)	0.421
**OMS**	0/1	1.0	
	2/3/4	1.1 (0.5-2.8)	0.788
**MMP-7**	<11.5 ng/mL	1.0	
	?11.5 ng/mL	2.1 (1.1- 4.4)	0.035
**Hemoglobin**	Normal	1.0	
	Abnormal	1.4 (0.7-2.6)	0.337

## Discussion

The prognosis of advanced bladder cancer is generally poor [22]. Therefore, new therapeutic strategies are needed to improve the outcome of patients with locally advanced or metastatic cancer. In this setting, the knowledge of prognostic determinants might be important for treatment planning and for patients¿ inclusion in clinical trials.

MMP-7 is overexpressed in many cancers. Previous studies have reported elevated MMP-7 concentration in urine samples and also in plasma samples from patients with bladder cancer. In urine samples no significant difference was detected in MMP-7 levels between bladder cancer patients and controls, suggesting that only MMP-7 plasma levels could be a putative biomarker for the diagnosis of bladder cancer [19]-[21]. Moreover, MMP-7 plasma levels may be used to identify patients at high risk of diseases progression [20],[21].

The aim of this study was to analyze the prognostic value of MMP-7 serum level particularly in advanced bladder cancer by quantifying MMP-7 concentration (using a sandwich fluoroimmunoassay) in serum samples from 56 patients with locally advanced or metastatic disease at time of MMP-7 analysis. Survival analysis made after categorization of MMP-7 serum levels into deciles indicated that concentrations higher than 11.5 ng/mL were associated with higher risk of poor prognosis (P?<?0.07). Multivariate analyses carried out using this cut-off and Cox proportional hazards regressions with a stepwise selection procedure to investigate the association with known prognostic factors showed that MMP-7 serum level is a strong independent prognostic factor of overall survival (HR?=?2.1, 95% CI 1.1-4.5, p?=?0.035). Our findings are consistent with those reported by Szarvas et al who analyzed MMP-7 gene expression and serum level in 179 patients with urothelial bladder cancer (160 patients with BUCC and 19 controls). The study showed that MMP-7 was significantly higher in both tumor and serum samples from patients with metastatic BUCC than in those without known metastasis. Furthermore, both high tissue and serum levels were stage- and grade-independent risk factors for metastasis and cancer-related death [23].

Although, the power of our analysis was reduced due to the small number of patients, our results suggest that MMP-7 serum level may be a prognostic factor for patients with locally advanced and or metastatic bladder cancer. We defined a MMP-7 cut-off value (11.5 ng/mL) that clearly differentiates two groups of patients: the overall survival of patients with low MMP-7 serum level was 3 years versus only 1.3 year for patients with high MMP-7 serum level.

## Conclusions

Our results show that the MMP-7 serum concentration is an independent prognostic factor in patients with locally advanced and or metastatic bladder cancer. Further studies are needed to determine how MMP-7 can adequately assign patients to prognostic subgroups for different treatments and how it can be used in the design of clinical trials.

## Abbreviations

MMP-7: Matrix metalloproteinase-7

TRACE: Time resolved amplified cryptate emission

BUCC: Bladder urothelial cell carcinoma

TURBT: Transurethral resection of the bladder tumor

ECM: Extracellular matrix

HR: Hazards ratio

CI: Confidence interval

PS: Performance status

## Competing interests

GS and BD are employees of Cezanne SAS, (part of Thermo Fisher Scientific).

## Authors¿ contributions

ME collected and assembled the data, and drafted the manuscript. GDS developed the immunoassay and carried out the assays. ST performed the statistical analysis and drafted the manuscript. WJ provided study patients and material, collected and interpreted the data and helped to draft the manuscript. YL performed the statistical analysis. BD participated in the conception the immunoassay and design of the study and helped to draft the manuscript. SC participated in the conception and design of the study, provided study material, interpreted the data and helped to draft the manuscript. XR provided study patients and material, interpreted the data and helped to draft the manuscript. PJL participated in the conception and design of the study, interpreted the data and drafted the manuscript. All authors read and approved the final manuscript.
